# Progressive Depletion of B and T Lymphocytes in Patients with Ataxia Telangiectasia: Results of the Italian Primary Immunodeficiency Network

**DOI:** 10.1007/s10875-022-01234-4

**Published:** 2022-03-08

**Authors:** Emilia Cirillo, Agata Polizzi, Annarosa Soresina, Rosaria Prencipe, Giuliana Giardino, Caterina Cancrini, Andrea Finocchi, Beatrice Rivalta, Rosa M. Dellepiane, Lucia A. Baselli, Davide Montin, Antonino Trizzino, Rita Consolini, Chiara Azzari, Silvia Ricci, Lorenzo Lodi, Isabella Quinti, Cinzia Milito, Lucia Leonardi, Marzia Duse, Maria Carrabba, Giovanna Fabio, Patrizia Bertolini, Paola Coccia, Irene D’Alba, Andrea Pession, Francesca Conti, Marco Zecca, Claudio Lunardi, Manuela Lo Bianco, Santiago Presti, Laura Sciuto, Roberto Micheli, Dario Bruzzese, Vassilios Lougaris, Raffaele Badolato, Alessandro Plebani, Luciana Chessa, Claudio Pignata

**Affiliations:** 1 Department of Translational Medical Sciences, Pediatric Section, Federico II University of Naples, via S. Pansini, 5-80131 Naples, Italy; 2 Department of Educational Sciences, University of Catania, Catania, Italy; 3 Department of Clinical and Experimental Sciences, University of Brescia and Department of Pediatrics, ASST-Spedali Civili Di Brescia, Brescia, Italy; 4 Unit of Immunology and Infectious Diseases, Academic Department of Pediatrics, Bambino Gesù Children’s Hospital, Rome, Italy; 5 Departments of Pediatrics, Fondazione IRCCS Ca’Granda Ospedale Maggiore Policlinico, Milan, Italy; 6 Division of Pediatric Immunology and Rheumatology, Department of Public Health and Pediatrics Regina Margherita Children Hospital, University of Turin, Turin, Italy; 7 Department of Pediatric Hematology and Oncology, ARNAS Civico Di Cristina and Benfratelli Hospital, Palermo, Italy; 8 Section of Pediatrics Immunology and Rheumatology, Department of Pediatrics, University of Pisa, Pisa, Italy; 9 Division of Pediatric Immunology, Department of Health Sciences, University of Florence and Meyer Children’s Hospital, Florence, Italy; 10 Department of Molecular Medicine, Sapienza University of Rome, Rome, Italy; 11 Department of Pediatrics, Policlinico Umberto I, Sapienza University of Rome, Rome, Italy; 12 Department of Internal Medicine, Fondazione IRCCS Ca’ Granda Ospedale Maggiore Policlinico, Milan, Italy; 13 Pediatric Hematology Oncology Unit, Azienda Ospedaliero Universitaria of Parma, Parma, Italy; 14 Division of Pediatric Hematology and Oncology, Ospedale G. Salesi, Ancona, Italy; 15 Unit of Pediatrics, IRCCS Azienda Ospedaliero-Universitaria, Bologna, Italy; 16 Pediatric Hematology/Oncology, Fondazione IRCCS Policlinico San Matteo, Pavia, Italy; 17 Department of Medicine, University of Verona, Verona, Italy; 18 Department of Public Health, Federico II University of Naples, Naples, Italy; 19 Sapienza University Foundation, Roma, Italy

**Keywords:** Ataxia telangiectasia, primary immunodeficiency, T lymphocytes, B lymphocytes, lymphopenia, genotype

## Abstract

Ataxia telangiectasia (AT) is a rare neurodegenerative genetic disorder due to bi-allelic mutations in the *Ataxia Telangiectasia Mutated* (*ATM*) gene. The aim of this paper is to better define the immunological profile over time, the clinical immune-related manifestations at diagnosis and during follow-up, and to attempt a genotype–phenotype correlation of an Italian cohort of AT patients. Retrospective data of 69 AT patients diagnosed between December 1984 and November 2019 were collected from the database of the Italian Primary Immunodeficiency Network. Patients were classified at diagnosis as lymphopenic (Group A) or non-lymphopenic (Group B). Fifty eight out of 69 AT patients (84%) were genetically characterized and distinguished according to the type of mutations in truncating/truncating (TT; 27 patients), non-truncating (NT)/T (28 patients), and NT/NT (5 patients). In 3 patients, only one mutation was detected. Data on age at onset and at diagnosis, cellular and humoral compartment at diagnosis and follow-up, infectious diseases, signs of immune dysregulation, cancer, and survival were analyzed and compared to the genotype. Lymphopenia at diagnosis was related per se to earlier age at onset. Progressive reduction of cellular compartment occurred during the follow-up with a gradual reduction of T and B cell number. Most patients of Group A carried bi-allelic truncating mutations, had a more severe B cell lymphopenia, and a reduced life expectancy. A trend to higher frequency of interstitial lung disease, immune dysregulation, and malignancy was noted in Group B patients. Lymphopenia at the onset and the T/T genotype are associated with a worst clinical course. Several mechanisms may underlie the premature and progressive immune decline in AT subjects.

**Supplementary Information:**

The online version contains supplementary material available at 10.1007/s10875-022-01234-4.

## Introduction

Ataxia telangiectasia (AT) (http://omim.org/entry/208900) is a rare genetic multisystem disorder of childhood due to mutations in the *Ataxia Telangiectasia Mutated* (*ATM*) gene. To date, over 600 distinct *ATM* mutations have been reported (www.hgmd.cf.ac.uk/ac/gene.php?gene=ATM) [1, 2]. Different types of *ATM* gene mutations are associated to different forms of the disease. Truncating mutations are related to the classic form of AT and are associated to the complete absence of the protein function. Splice site mutations or missense mutations allow for some residual ATM kinase activity, and this causes milder forms of AT [3–5]. ATM protein exerts a central role in the signal-transduction pathway activated by DNA double-strand breaks (DSBs); therefore, AT is considered the prototype of the DNA-repair defect syndromes [6, 7]. Of note, gene rearrangements and DSBs repair by ATM are required for a proper maturation of either T or B cells [2]. Moreover, in B cells the absence of a functional ATM protein leads to a defect in class switch recombination (CSR), thus supporting a role of ATM also in this process [5, 8].

The clinical phenotype of AT mainly includes oculocutaneous telangiectasia; immunodeficiency; growth failure; autoimmune diseases; endocrine, hepatic, and cardiovascular disorders; high incidence of neoplasms; and hypersensitivity to ionizing radiations [2]. The hallmark of the disease is a progressive neurological degeneration, with ataxia and movement disorders, due to selective depletion of Purkinje cells, invariably confining the patients to wheelchair with great worsening of the quality of life [9–11]. Both humoral and cellular immune system may be impaired, being characterized by inadequate production of immunoglobulins, T cell lymphopenia, and defective proliferative response to mitogens, predisposing to recurrent bacterial sinopulmonary infections [7, 11, 12].

The aim of this study is to better define the immunological features and the clinical immune-related manifestations at diagnosis and during follow-up of an Italian cohort of AT patients. Results are compared to the genotype.

## Methods

### Study Design, Patients, and Data Collection

This is a retrospective multicenter longitudinal clinical study involving data from 69 patients with AT diagnosed on the basis of the clinical criteria and alpha-fetoprotein (AFP) values, according to the recent World Health Organization (WHO) recommendations and the European Society for Immunodeficiencies (ESID) guideline [13–15]. Data were collected from the database (DB) of the Italian Primary Immunodeficiency Network (IPINet), entered from December 1984 to November 2019. Age at onset (AO) was defined as the age at the first documented AT-related symptoms, age at diagnosis (AD) as the age at clinical or molecular diagnosis of AT, while the follow-up period (Fup) was defined as the period starting from diagnosis until November 2019 or death of the patient. Long survivors were defined those AT patients with an age at death or at the time of the last Fup ≥ 25 years or those patients living with AT above the age of 25 years.

Data on lymphocyte counts, serum immunoglobulin levels (IgA, IgG, and IgM), and immunophenotype, including T cells (CD3^+^), helper T cells (CD3^+^CD4^+^), cytotoxic T cells (CD3^+^CD8^+^), naïve T cells (CD4^+^ or CD8^+^, CD45RA^+^), B cells (CD3^−^CD19^+^), and natural killer (NK) cells (CD3^−^CD16^+^/CD56^+^), evaluated through standard methods, were collected and compared to healthy age-matched controls [16]. Total IgA deficiency was defined as IgA serum levels lower than 7 mg/dL in patients older than 4 years, evaluated through nephelometry, while partial IgA deficiency was considered for values of serum IgA comprised between 7 mg/dL and ≤ 2 SD of normal values for age [17].

According to their genotype (type of mutations), patients carrying bi-allelic truncating mutations were indicated as T/T, while patients with at least one missense or splice site mutation not determining a stop codon were defined as T/NT. Patients with two non-truncating mutations were defined as NT/NT. For our purpose, we distinguished AT patients at diagnosis as lymphopenic (Group A) or non-lymphopenic (Group B). Either group was then analyzed for AO, AD, cellular and humoral compartment at diagnosis and Fup, infectious diseases, immune dysregulation as autoimmune cytopenia, diabetes, thyroiditis, cancer, and survival. Results were then compared to genotype. Chronic bronchopneumopathy was defined as cough for at least 8 weeks, 2 or more times per year or abnormal auscultation of the chest 3 or more times in a year, and/or the presence of non-resolving lobar consolidation, bronchiectasis, peribronchial wall thickening, and atelectasias at magnetic resonance imaging (MRI) or computed tomography (CT), when performed [18]. Records from radiographs, CT, or MRI scan were collected when available. Globally, 40% of the patients received a CT or MRI evaluation. When no imaging was available, patients were defined as affected by pneumonia according to clinical criteria and response to antibiotic treatment.

A written informed consent was obtained from the participants or parents/legal guardians. The study was conducted following the ethical principles of the Declaration of Helsinki, and the code of Good Clinical Practice. The study was approved by Institutional Ethics Committees (No. 628/2018).

#### Statistical Analysis

Data were shown as mean ± SD, median and range, or frequencies (number of cases) and percentages as appropriate. Student’s *t*-test was used to compare means for continuous variables. Chi-square test or Fisher’s exact test was used for categorical variables. Comparisons between groups were performed using the non-parametric Mann–Whitney test for quantitative variables. All *P*-values are two-sided and values ≤ 0.05 were considered significant. The calculations were performed using GraphPad Prism 5.0. Survival curves were estimated according to the Kaplan–Meier method and compared with the Mantel-Cox test; significance for survival curves was set at *P* ≤ 0.05.

## Results

### Patients’ Characteristics

The study included 69 children and adults with AT (29 males); mean age at the time of the study ± SD was 16.2 ± 8.7 years (range: 2.6–41.6 years), with a mean Fup period of 6 years per patient and cumulative Fup data for 252 patient-years. Data was only available at diagnosis or at Fup for 4 and 5 patients, respectively.

Almost all patients (*n* = 64, 92.8%) were of Italian origin, while the remaining 5 (7.2%) were of a different geographic origin (3 from North Africa, 2 from East Europe).

Overall, mean AO ± SD was 25.6 ± 21.5 months, and mean AD ± SD was 72.6 ± 60.4 months. Nine subjects had a late**r** age of onset (> 36 months) and, within this subgroup, the mean AO ± SD was 73 ± 11 months.

The majority of the subjects had a sporadic form, while affected siblings were identified in 7 families. At the end of the Fup, 11 patients were dead at a mean age ± SD of 22.4 ± 6.5 years. Causes of death were severe pneumonia (*n* = 6), pneumonia + malignancy (*n* = 2), and malignancy (*n* = 3). Eight subjects were long survivors (mean age ± SD: 31.1 ± 5.71 years, range 25.6–41.6).

### Genomic Features

Fifty eight out of 69 AT patients (84%) were genetically characterized, while the remaining were diagnosed only on the basis of clinical criteria and AFP values. Most patients were compound heterozygotes, while 17 carried homozygous mutations. In three subjects only one pathogenic variant was identified. A high genomic heterogeneity was observed: recurrent mutations c.7517del4 and c.3576G > A were identified in 6 and 5 subjects, respectively. Overall, 27 subjects carried null mutations in both alleles (T/T), 23 patients were T/NT, and 5 were NT/NT. Fourteen subjects included in the cohort were defined genotypically unclassified since molecular test was not available at the time of diagnosis (*n* = 11) or only one pathogenic variant was identified (*n* = 3). Within the Group T/T, 8 subjects had nonsense mutations on both alleles, and 9 carried a nonsense mutation + another truncating variant (frameshift mutation or deletion). Frameshift mutations on both alleles or associated to a splice site mutation were detected in 4 and 3 subjects, respectively, while 3 subjects had splice site mutations on both alleles.

**Fig. 1:**
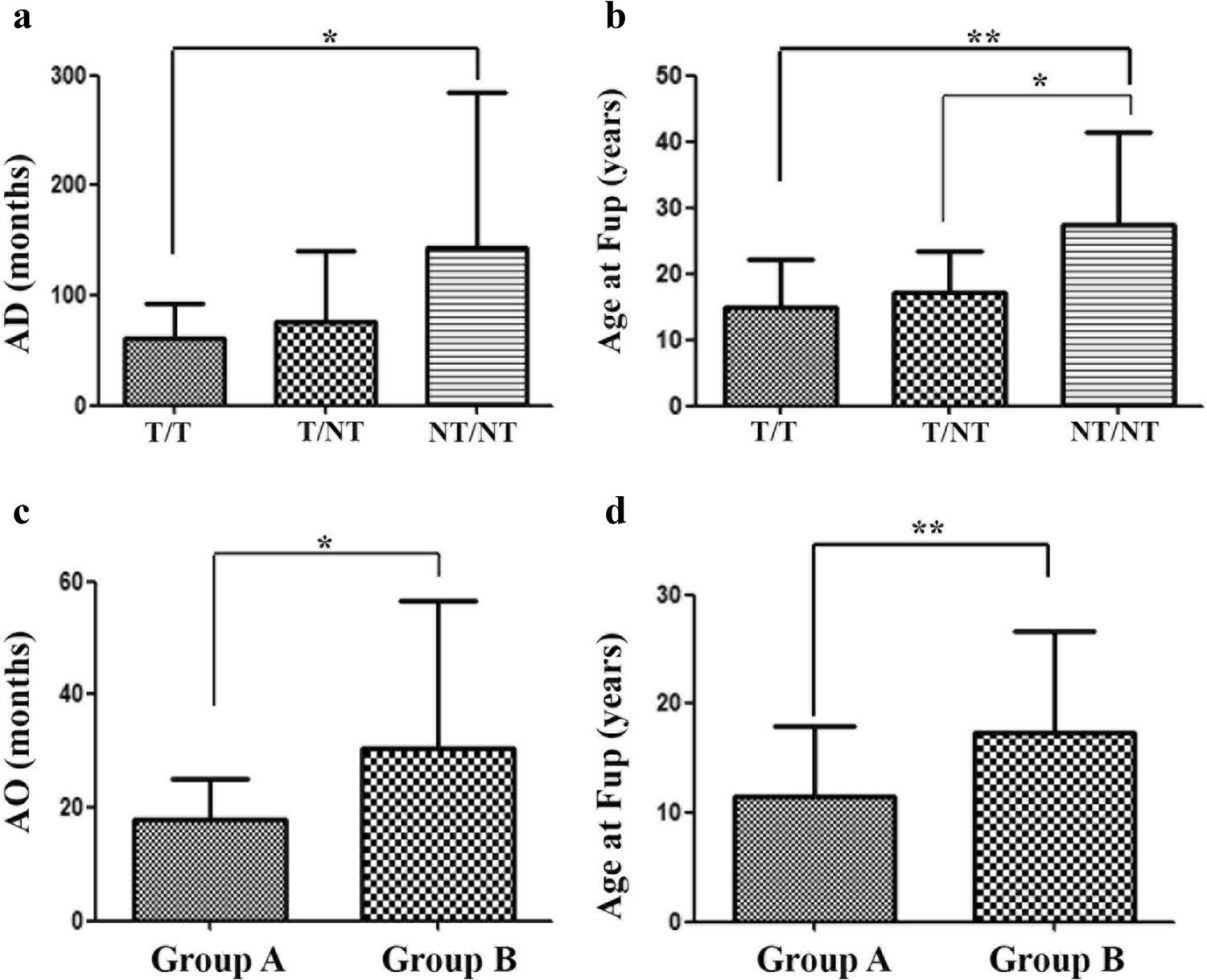
Demographic data of Italian AT cohort according to genotype and immunological status at diagnosis. **a** and **b** Age at diagnosis (AD) and at follow-up (Fup) was significantly lower in T/T patients compared to patients carrying both non-truncating mutations (NT/NT). A significant difference was also observed among patients carrying one non-truncating (T/NT) and those with 2 non-truncating variations (NT/NT). **c** and **d** Age at diagnosis (AD) and at follow-up (Fup) was significantly lower in lymphopenic patients (Group A) as compared to non-lymphopenic subjects (Group B)

AO was similar in all AT patients regardless to their genotype. On the contrary, as shown in Fig. 1a, AD was significantly lower in T/T patients as compared to NT/NT patients (mean ± SD, 61.4 ± 31.1 vs 143.2 ± 141.6 months, *P* = 0.01), indicating a more severe phenotype in this group. As shown in Fig. 1b mean age ± SD at last Fup was significantly lower in T/T when compared with NT/NT (mean ± SD, 14.9 ± 7.26 vs 27.4 ± 13.9 years, *P* = 0.0072). Mean age ± SD at last Fup was lower in Group T/NT carrying one non-truncating variant as compared to those with 2 non-truncating variants (17.21 ± 6.3 vs 27.4 ± 13.9, *P* = 0.033).

### Immunological Features

#### Humoral Compartment

Humoral compartment was evaluated in all patients (Table 1). At diagnosis, 12.3% (8/65) of the patients showed low IgG serum levels with at least one additional Ig class reduction (IgA or IgM), 9.2% (6/65) had isolated low IgG levels, and 4.6% (3/65) had pan-hypogammaglobulinemia. A selective IgAD was observed in 35.3% (23/65); among them 6.1% had a partial IgAD. High IgM or IgA levels were found in 12.3% (8/65) and 3.1% (2/65) of the patients, respectively. Specific antibody levels to protein-based or live vaccine (tetanus, diphtheria, or measles) were evaluated in 35 patients. Protective titles were found in 24 (68.6%) of them. One of 11 patients with low levels also had pan-hypogammaglobulinemia, 3 had reduced IgG and IgA levels, 1 of them also having high IgM levels, and 2 had SIgAD. All but one were lymphopenic, with reduction of CD3^+^, CD4^+^, and CD8^+^ T cells. Four patients had a B cell lymphopenia. During the Fup, 20 patients required Ig replacement therapy.

**Table 1:** Humoral defects in the Italian AT cohort

	Diagnosis	T/T	T/NT-NT/NT	Unclassified	*P* value	Fup	T/T	T/NT-NT/NT	Unclassified	*P* value
	No. (%)	No. (%)	No. (%)			No. (%)	No. (%)	No. (%)		
Normal immunoglobulin levels	15 (23)	4 (16)	7 (26.9)	4 (28.6)	0.49	13 (22.8)	5 (22.7)	8 (30.7)	0 (0)	0.74
Panhypogammaglobulinemia	3 (4.6)	2 (8)	1 (3.8)	0 (0)	0.61	20 (35.1)	9 (40.9)	8 (30.7)	3 (33)	0.55
IgG with IgA or IgM deficiency	8 (12.3)	2 (8)	5 (19.2)	1 (7.1)	0.41	3 (5.3)	1 (4.5)	2 (7.7)	0 (0)	1.00
Isolated IgG deficiency	6 (9.2)	1 (4)	2 (7.6)	3 (21.4)	1.00	5 (8.8)	0 (0)	3 (11.5)	2 (22.2)	0.24
Selective IgAD	23 (35.3)	11 (44)	7 (26.9)	5 (35.7)	0.24	7 (12.3)	3 (13.6)	2 (7.7)	2 (22.2)	0.64
Total IgAD	19 (29.2)	9 (36)	6 (23.1)	4 (28.6)	0.36	6 (10.5)	1 (4.5)	3 (11.5)	2 (22.2)	0.61
Partial IgAD	4 (6.1)	2 (8)	1 (3.8)	1 (7.1)	0.61	1 (1.7)	1 (4.5)	0 (0)	0 (0)	0.46
High IgM	8 (12.3)	2 (8)	4 (15.4)	2 (14.3)	0.66	6 (10.5)	3 (13.6)	2 (7.7)	1(11.1)	0.64
Isolated high IgM	1 (1.5)	0 (0)	1 (3.8)	0 (0)	1.00	3 (5.3)	2 (9.1)	1 (3.8)	0 (0)	0.60
Isolated high IgA	2 (3.1)	2 (8)	0 (0)	0 (0)	0.23	3 (5.3)	2 (9.1)	1 (3.8)	0 (0)	0.60
Total	65	25	26^ǂ^	14		57	22	26^ǂ^	9	

*IgAD* IgA deficiency, *T/T* bi-allelic truncating mutations, *T/NT* 1 non-truncating mutation, *NT/NT* bi-allelic non-truncating mutations

^ǂ^5 NT/NT subjects were included

*P* value: T/T vs T/NT-NT/NT

Among patients who were diagnosed as selective IgAD at diagnosis, 2 (1 partial IgAD) spontaneously reached normal IgA values, 1 developed high IgM levels without IgG reduction, 3 developed a pan-hypogammaglobulinemia requiring Ig replacement therapy, and 4 required replacement therapy due to recurrent infections.

No significant difference in humoral immunity was observed between Group A and Group B, neither at diagnosis nor during Fup. No difference in the spectrum of humoral alterations was found among subjects with bi-allelic non-truncating variants and the other groups (data not shown).

#### Cellular Compartment

As shown in Table S1, at diagnosis 41.5% (27/65) of AT patients were lymphopenic, as compared to age-matched reference values [16], and assigned to Group A, while 58.5% (38/65) patients had normal or even increased lymphocyte counts (Group B). As depicted in Fig. 1c, AO was significantly lower in Group A than in Group B (mean ± SD, 17.9 ± 7.16 vs 30.6 ± 25.9, *P* = 0.023). No difference was noted among these 2 groups in AD. Age of patients at Fup (Fig. 1d) was significantly lower in Group A when compared to Group B (mean + SD, 11.45 ± 5.52 vs 17.3 ± 9.35 years, *P* = 0.007).

According to the molecular data available at diagnosis in 51 subjects (T/T: 25, T/NT: 21, NT/NT: 5), we found that 52% (13/25) of T/T patients and 30.8% (8/26) of T/NT belonged to Group A (*P* = 0.08). All 5 NT/NT patients belonged to Group B.

T and B cell compartments were evaluated at diagnosis in 63 patients. Eleven subjects (17.4%) had a close to normal T and B cell compartment (T/T: 3, T/NT: 5, NT/NT: 2, unclassified: 1). All of them belonged to Group B. CD3^+^ absolute number was low in 61.9% (39/63; 26 belonged to Group A) of patients. In particular, 2 of them, who carried the 5932G > T/8278C > T and 7240del5/7408 T > G mutations, respectively, had less than 300 cells/μL, while 17 (T/T: 6, T/NT: 8, NT/NT: 1, unclassified: 3) had values between 300 and 500 cells/μL. Absolute CD4^+^ and CD8^+^ counts were low in 68.2% (43/63; 26 belonged to Group A) and 49.2% (31/63; 18 belonged to Group A) of the subjects, respectively. In subjects with abnormal CD4 values, mean counts were 368.3 ± 195.7 cells/μL, and 11 of them (T/T: 5, T/NT: 5, unclassified: 1) had a marked reduction of absolute number of CD4^+^ T cells (< 200 cells/μL). Percentage of naïve CD4^+^ and CD8^+^ T cells were evaluated in 27 subjects at the diagnosis and were found lower than normal age value in the majority of them (24/27, 88.8%). In particular, 7 patients had a percentage of CD4^+^ naïve cells lower than 10%. An expansion of CD3^+^CD4^−^CD8^−^ (DNeg) (> 5% of the circulating CD3^+^ T cells) T cells, potentially involved in the pathogenesis of different immune-mediated diseases, was found in 29 subjects at diagnosis, ranging from 6 to 31%.

Absolute number of CD19^+^ cells was reduced in 33.3% of the patients (21/63; 12 belonged to Group A). T/T patients showed lower B cell counts as compared to T/NT (mean ± SD 158 ± 135 vs 248 ± 146 cells/μL; *P* = 0.049). In NT/NT subjects, the number of CD19^+^ cells were not different from that observed in the T/NT (245.6 ± 42.54 vs 281.9 ± 40.27; *P* = 0.67)**.**

Of the cohort of 65 patients enrolled at the diagnosis, no data on NK cells were available for 20 patients. In all the other patients but one, NK cells were normal (30/45, 66.6%) or increased (14/45, 31.1%).

Data on proliferative response to phytohemagglutinin (PHA) were available at diagnosis in 25 patients. Thirteen of them (52%), subjects evaluated at a mean age ± SD of 56.1 ± 31.2 months, had a response < 30% compared to controls and, among them, 6 had a severe functional defect (PHA < 10% of control). No difference was observed in this small group among patients with different genotype (Table S1). Moreover, no difference was observed among patients who belonged to Group A or B. However, the three subjects with NT/NT mutations showed a higher proliferative response to PHA; in particular, the two homozygous patients for the variant c.3576G > A had a near-normal proliferative response.

During the Fup, lymphocyte counts and T and B immunophenotype were available for 58 and 54 patients, respectively. On the whole cohort, most of the lymphopenic patients remained lymphopenic, while within the non-lymphopenic group at onset, 11/38 patients (29%) became lymphopenic. Absolute values of total lymphocytes, CD3^+^, and CD8^+^ cells did not change during the Fup (Table 2). In contrast, in the whole group of patients a significant decrease in the absolute number of CD19^+^ cells (*P* = 0.03) and an increase of CD4^+^ (*P* = 0.05) were observed. No correlation with the genotype was found. Among T/T and T/NT patients, the percentage of subjects who had at diagnosis CD3^+^, CD4^+^, CD8^+^, and CD19^+^ cells lower than age-matched controls did not change over the time.

**Table 2:** Comparison of lymphocyte subpopulations of the whole cohort between diagnosis and Fup

	Diagnosis	Fup	*P* value
Lymphocytes cells/mL (mean ± SD)	1639 ± 1005	1757 ± 1933	0.66
CD3^+^ cells/mL (mean ± SD)	766.9 ± 72.17	858.1 ± 85.08	0.41
CD4^+^ cells/mL (mean ± SD)	368.3 ± 24.86	493.1 ± 60.95	**0.05**
CD8^+^ cells/mL (mean ± SD)	279.6 ± 33.41	299.9 ± 34.48	0.67
CD19^+^ cells/mL (mean ± SD)	210.7 ± 19.60	143.2 ± 24.23	**0.03**
CD3^−^CD16^+^/CD56^+^ cells/mL (mean ± SD)	522 ± 435.1	/	

Bold indicates statistical significance (*P* ≤ 0.05)

Unfortunately, only limited data on CD3^−^CD16^+^/CD56^+^ cells were available during the Fup; thus, further considerations were not possible.

**Fig. 2:**
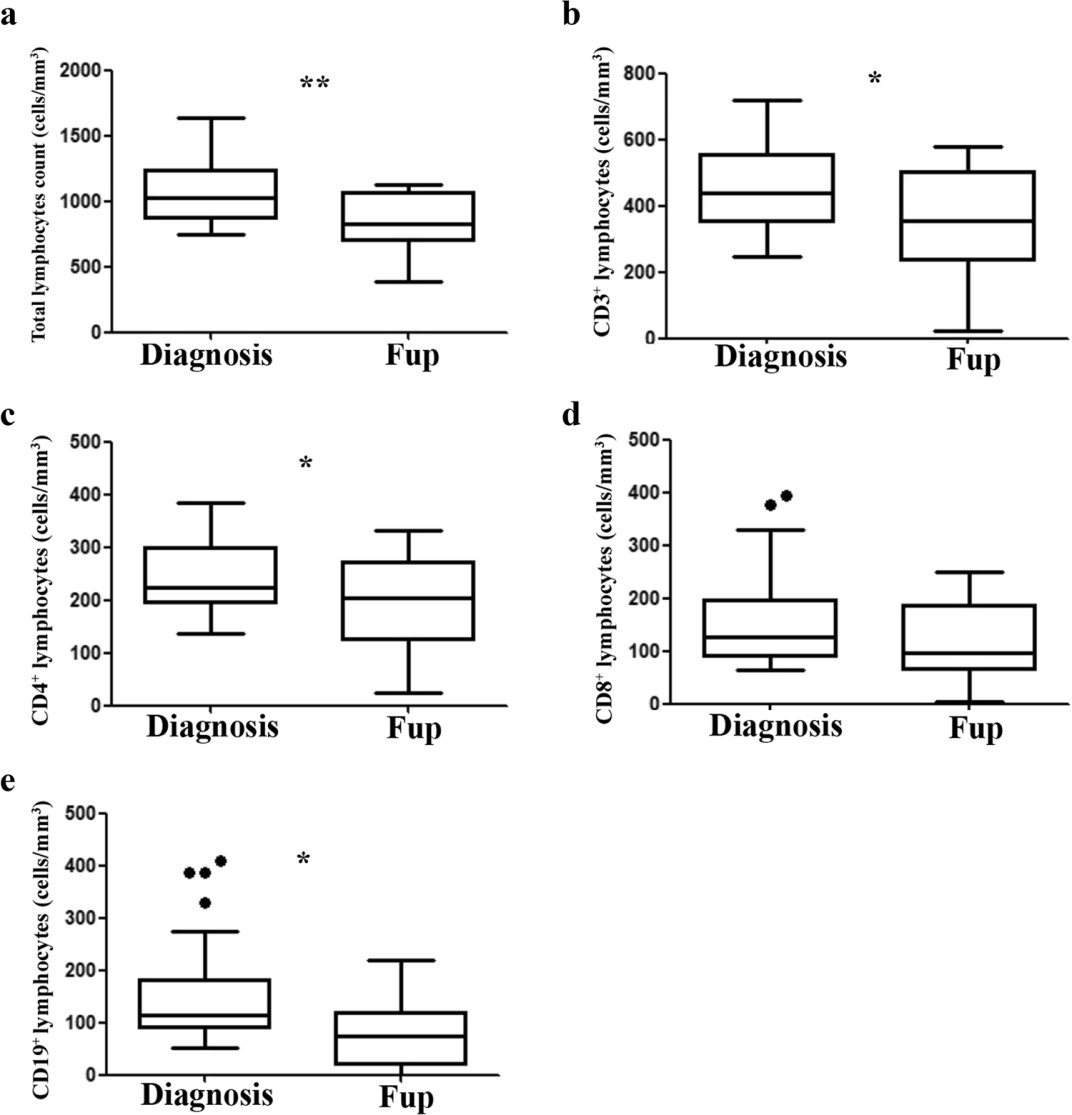
Dynamic changes of T and B immunophenotype in Group A patients during follow-up (Fup). **a** Lymphocyte counts. **b–e** CD3^+^, CD4^+^, CD8^+^, and CD19^+^ cell absolute counts. *P < 0.05 and **P < 0.01

We next evaluated in the Group A patients the behavior over the time of lymphocyte counts and subsets. As shown in Fig. 2, a progressive and statistically significant decrease of total lymphocyte counts, CD3^+^, CD4^+^, and CD19^+^ lymphocytes was observed during disease progression. No change in the CD8^+^ cells was noted. Of note, no difference was appreciated in all the variables over time in the Group B patients, while among non-lymphopenic patients who showed a progression to lymphopenia, a statistically significant reduction was observed only for CD19^+^ cells (mean ± SD at diagnosis 248 ± 55 vs 86.6 ± 19.5 at Fup, *P* = 0.015).

### Intrafamilial Immunological Comparison

An intrafamilial comparison of the immunological and clinical phenotype in 7 families with 2 affected siblings was performed (Table 3). Despite their common genetic background, clinical heterogeneity and a difference in immunological profiles were evident among these patients. AO was similar in the affected patients, except in the sibling pairs E47-48 in which the brother had a later onset. Three siblings were discordant for Group A and B classification, and in 2 sibling pairs (C36-37 and E47-48) a discordance in the reduction of CD3^+^, CD4^+^, and CD8^+^ cells was also noted. In the humoral compartment evaluation, a discordance for hypogammaglobulinemia with high IgM was observed in A8-9 and G64-65 sibling pairs. Patient F53 had a severe hypogammaglobulinemia, while his sister only showed an IgA deficiency.

**Table 3:** Phenotype and genotype of Italian familial AT patients

Patient	Sex	AO (months)	AD (months)	Age at last Fup (years)	Genotype	Group	Infections	Immune dysregulation	Cancer	Cellular abnormalities	Humoral abnormalities	Status
A8	F	72	310	41.6	c.3576G > A/c.3576G > A	B — NT/NT (20%)	+	+	−	Low CD3^+^	Low IgG, high IgM	Alive
A9	F	72	283	39	c.3576G > A/c.3576G > A	B — NT/NT (20%)	−	+	+	N	N	Alive
B15	F	24	100	12	c.7517del4/c.5692C > T	A — T/T (0%)	+	−	−	Low CD3^+^, CD4^+^, CD8^+^	N	Alive
B16	M	12	50	8.6	c.7517del4/c.5692C > T	B — T/T (0%)	+	−	+	Low CD3^+^, CD4^+^	Low IgG, IgA	Dead
C36	M	12	70	Unknown	c.7240del4/c.7408 T > G	B — T/NT (50%)	−	−	−	N	N	Unknown
C37	F	12	29	17	c.7240del4/c.7408 T > G	A — T/NT (50%)	+	−	+	Low CD3^+^, CD4^+^, CD8^+^, CD19^+^	SIgAD	Alive
D42	F	12	41	6	Not available	A	−	−	+	Low CD3^+^, CD4^+^, CD8^+^	Low IgG, IgA	Alive
D43	F	12	13	3	Not available	A	−	−	−	Low CD3^+^, CD4^+^	Low IgG, IgA	Alive
E47	F	12	110	23	c.3111delT/c.3576G > A	B — T/NT	−	+	−	N	N	Alive
E48	M	60	72	17	c.3111delT/c.3576G > A	B — T/NT	−	−	−	Low CD3^+^, CD4^+^, CD8^+^	N	Alive
F52	M	35	77	13.6	c.3894_3895insT/c.3894_3895insT	B — T/T	−	−	−	Low CD8^+^	Pan-hypogammaglobulinemia	Alive
F53	F	23	30	8.6	c.3894_3895insT/c.3894_3895insT	A — T/T	−	−	−	Low CD3^+^, CD4^+^, CD8^+^	SIgAD	Alive
G64	F	4	79	18.8	c.6679C > T/c.8484delA	A — T/NT (10%)	+	−	−	Low CD3^+^, CD4^+^, CD8^+^, CD19^+^	Low IgG, high IgM	Alive
G65	M	24	39	15.8	c.6679C > T/c.8484delA	A — T/NT (10%)	−	−	−	Low CD3^+^, CD4^+^, CD8^+^	Low IgG	Alive

*AO* age at onset, *AD* age at diagnosis, *T/T* bi-allelic truncating mutations, *T/NT* at least one non-truncating mutation, *NT/NT* non-truncating mutations on both alleles, *A* lymphopenic, *B* non-lymphopenic, *SIgAD* selective IgA deficiency, *N* normal. The brackets show the percentage of residual ATM protein, when available

During the Fup, 3 patients from 3 unrelated families developed malignancy, differently from their siblings despite a similar age.

### Infections, Immune Dysregulation, and Cancer

**Fig. 3:**
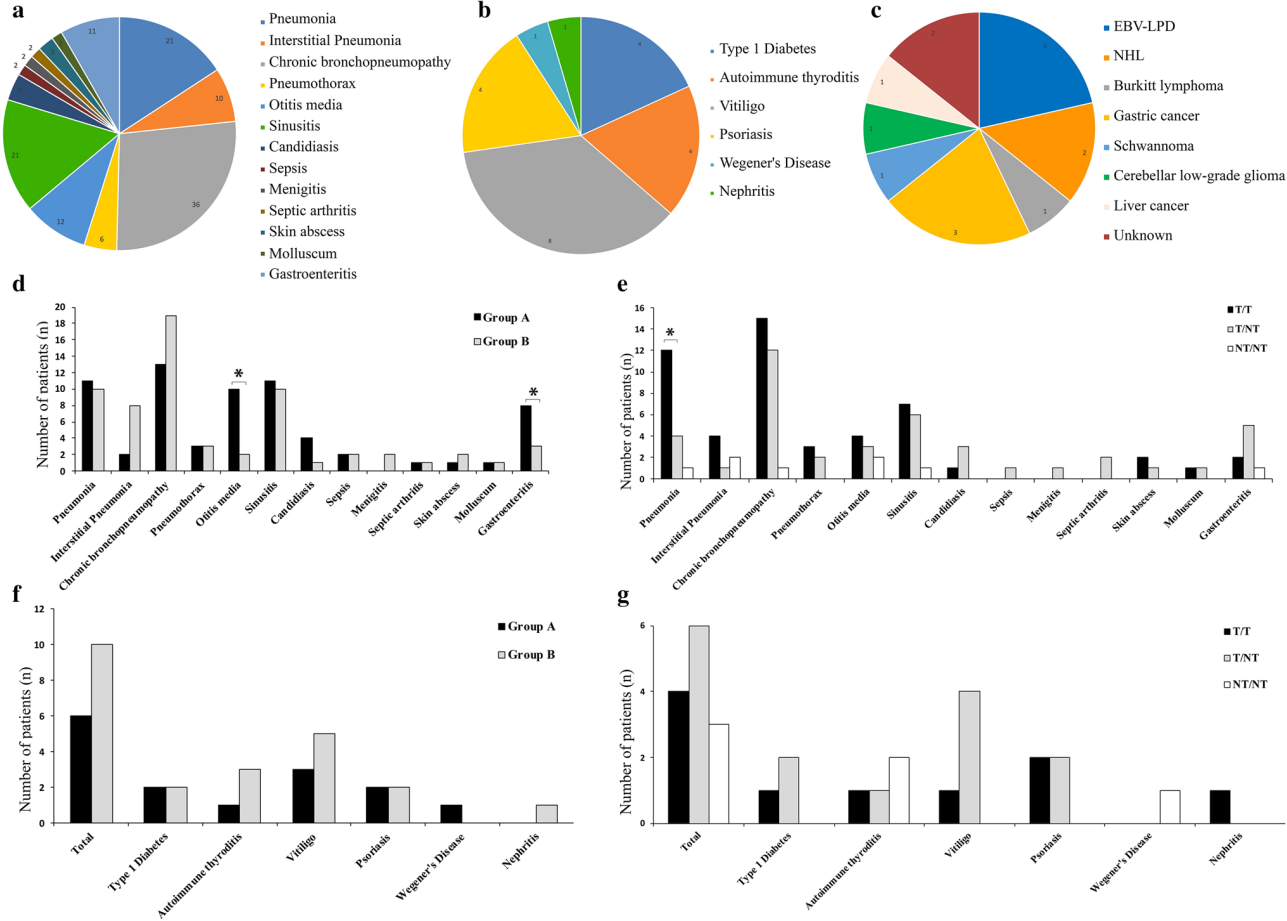
Clinical phenotype of the cohort. **a–c** Number of subjects in the whole cohort showing infections, immune dysregulation, and malignancies. **d** Comparison of infectious manifestations among patients belonging to Group A and B. **e** Comparison of infectious manifestations among T/T, T/NT, and patients carrying both non-truncating mutations (NT/NT). **f** Comparison of immune dysregulation events among patients belonging to Group A and B. **g** Comparison of immune dysregulation among T/T, T/NT, and NT/NT patients. *T/T* bi-allelic truncating mutations, *T/NT* at least one non-truncating mutation, *NHL* non-Hodgkin lymphoma. *P < 0.05 and **P < 0.01

In Fig. 3a–c, the number of patients with infections, autoimmunity, or malignancies were reported. Infections were reported in the majority of the cohort (88%; 61/69). Signs and symptoms of a respiratory tract involvement were the most frequent clinical features (Fig. 3a), with pneumonias recorded in 30.4% (21/69) of the patients, mean number of 0.16 episode per patient-year; interstitial pneumonia in 14.5% (10/69), mean number of 0.039 episode per patient-year; chronic bronchopneumopathy in the 52.2% (36/69), mean number of 0.29 episode per patient-year; otitis media in 17.4% (12/69), mean number of 0.055 episode per patient-year; and sinusitis in 24.6% (17/69), mean number of 0.14 episode per patient-year. Four of the 9 patients with ILD had evidence of an infectious process or history of recurrent pneumonia due to common bacterial pathogens. Two further subjects had cancer or an autoimmune disorder, respectively.

Of note, during the Fup 8.7% (6/69) of AT patients developed a pneumothorax, mean number of 0.0238 episode per patient-year. A positive history of gastroenteritis was recorded in 15.9% (11/69) of the patients, mean number of 0.092 episode per patient-year. Skin infections, as abscesses or molluscum contagiosus, were reported in 4.3 (3/69) and 2.9% (2/69) of the subjects, respectively, mean number of 0.011 and 0.007 episode per patient-year. Invasive infections, including sepsis, arthritis, and meningitis, were reported in 8.7% (6/69) of the patients, mean number of 0.023 episode per patient-year. Finally, mucocutaneous candidiasis was documented in 5 patients (7.2%), mean number of 0.0198 episode per patient-year.

As for the recurrence of infections, 5 (7.2%) patients suffered from recurrent otitis (> 2 episodes/year), and 15 (21.7%) patients suffered from recurrent pneumonia (1 episodes/year for > more than 1 year), of whom 2 of them were interstitial, and 2 (2.89%) patients experienced two episodes of mucocutaneous candidiasis. Four (5.8%) patients suffered from upper airway infections as sinusitis (> 3 episodes) and 5 (7.24%) exhibited more than 2 episodes of gastroenteritis.

As shown in Fig. 3d, a comparison of clinical manifestations between Group A and B patients revealed that otitis media, mucocutaneus candidiasis, and gastrointestinal infections were slightly more frequent in lymphopenic subjects, while interstitial pneumonia and chronic bronchopneumopathy were more frequent in non-lymphopenic subjects, even though the difference was not statistically significant. Group A patients had a trend of higher number of per-patient infectious episodes, when compared to Group B (2.39 vs 1.56 episodes/patients, *P* = 0.83). When we divided the patients according to T and B cell subsets, we found that pneumonia was statistically more frequent in patients with low CD3^+^ (16/39, 41% vs 2/24, 8.3%, *P* = 0.0085) and CD4^+^ (17/43, 39.5% vs 1/18, 5.5%, *P* = 0.012) T cells. Patients with low CD4^+^ also had a higher prevalence of gastroenteritis (11/43, 25.6% vs 0/18, *P* = 0.0246). No correlation was found concerning invasive or severe infections, including meningitis, sepsis, or septic arthritis among the different subgroups. We also compared the risk of infection or autoimmunity according to naïve T cell number. No significant difference was found. When T/T and T/NT patients were compared, pneumonia was more frequent in the former (*P* = 0.027) (Fig. 3e), and more in general, a trend to a higher number of infectious episodes per patient was noted in T/T (2.01 and 1.61, respectively; *P* = 0.40). Of note 2/5 patients carrying both non-truncating mutations developed interstitial lung disease (ILD) during the Fup. As for the association with cellular immunity abnormalities, when the proliferative response data were available, we found a significant increase of pneumonia (7/13, 53.8% vs 1/12, 8.3%, *P* = 0.030) and bronchopneumopathy (10/13, 76.9% vs 2/12, 16.6%, *P* = 0.0048) in patients with PHA response < 30% of controls as compared with patients with near-normal response. Lastly, we evaluated infectious risk according to both genotype and the immunological status at diagnosis. In particular, 13 patients were assigned to T/T-Group A, 11 to T/T-Group B, 7 to T/NT-Group A, 13 to T/NT-Group B, and 5 to NT/NT-Group B. No significant difference was found (data not shown). However, the number of infectious episodes per patient was significantly lower in T/NT-Group B patients (1.46 ± 0.39) when compared to both T/T-Group B (4.89 ± 1.43, *P* = 0.0035) or T/NT-Group A (5.71 ± 1.41, *P* = 0.0015).

Regarding the immune dysregulation, 23.1% (16/69) of the patients showed autoimmune disorders (Fig. 3b). Endocrine organs and skin were the main target of autoimmunity, as patients had autoimmune thyroiditis (5.8%, 4/69), type 1 diabetes mellitus (5.8%, 4/69), vitiligo (11.6%, 8/69), and psoriasis (5.8%, 4/69). One patient developed Wegener granulomatosis and one patient autoimmune nephritis. Cutaneous granuloma was reported in 3 patients.

Interestingly, autoimmunity was more frequent, although not statistically significant, in T/NT and in Group B patients (Fig. 3f,g). When the immunological phenotype was compared with autoimmune disorders, these were more frequent in patients with low CD19^+^ cells (9/21, 42.8% vs 6/41, 14.6%, *P* = 0.0261). Seven patients (43.7%) also had an increased number of DNeg T cells.

Fourteen subjects developed malignancy with a male/female ratio of 0.55. The majority of patients manifested hematopoietic neoplasia including EBV-related lymphoproliferative disorders (4.34%, 3/69), non-Hodgkin lymphoma (NHL) (2.9%, 2/69), and Burkitt lymphoma (1.4%, 1/69) (Fig. 3c). One patient affected by NHL also suffered from a schwannoma. Solid cancer as gastric carcinoma (4.34%, 3/69), cerebellar low-grade glioma (1.4%, 1/69), and liver cancer (1.4%, 1/69) were reported. In 2/14 no data on the type of malignancy were available. A trend to a higher frequency of cancer among Group B patients was noted (26.3%, 10/38 vs 16.6%, 4/27 of Group A), with a relative risk of 1.77 (95% CI 0.622 to 5.0729, *P* = 0.283). When we evaluated the incidence of cancer according to both genotype and the immunological status at diagnosis we found that cancer occurred in 7% of patients who belonged to T/T-Group A (1/13) and in 45.4% (5/11) of patients who belonged to T/T-Group B (*P* = 0.06). There was not any correlation between the major lymphocyte subsets and malignancies.

When we compared clinical manifestations with serum immunoglobulin levels, we found that patients with low IgG had a statistically significant increase of gastroenteritis (8/23, 34.8% vs 3/42, 7.1%, *P* = 0.011). No further difference was observed for the other clinical features, including severe or invasive infections, autoimmunity, or malignancy. Cancer occurred more frequently in patients with T cell abnormal functionality, even though the difference was not statistically significant (6/13, 46% vs 1/12, 8.3%, *P* = 0.073). No correlation was observed among autoimmune manifestations and PHA response.

### Survival Analysis

**Fig. 4:**
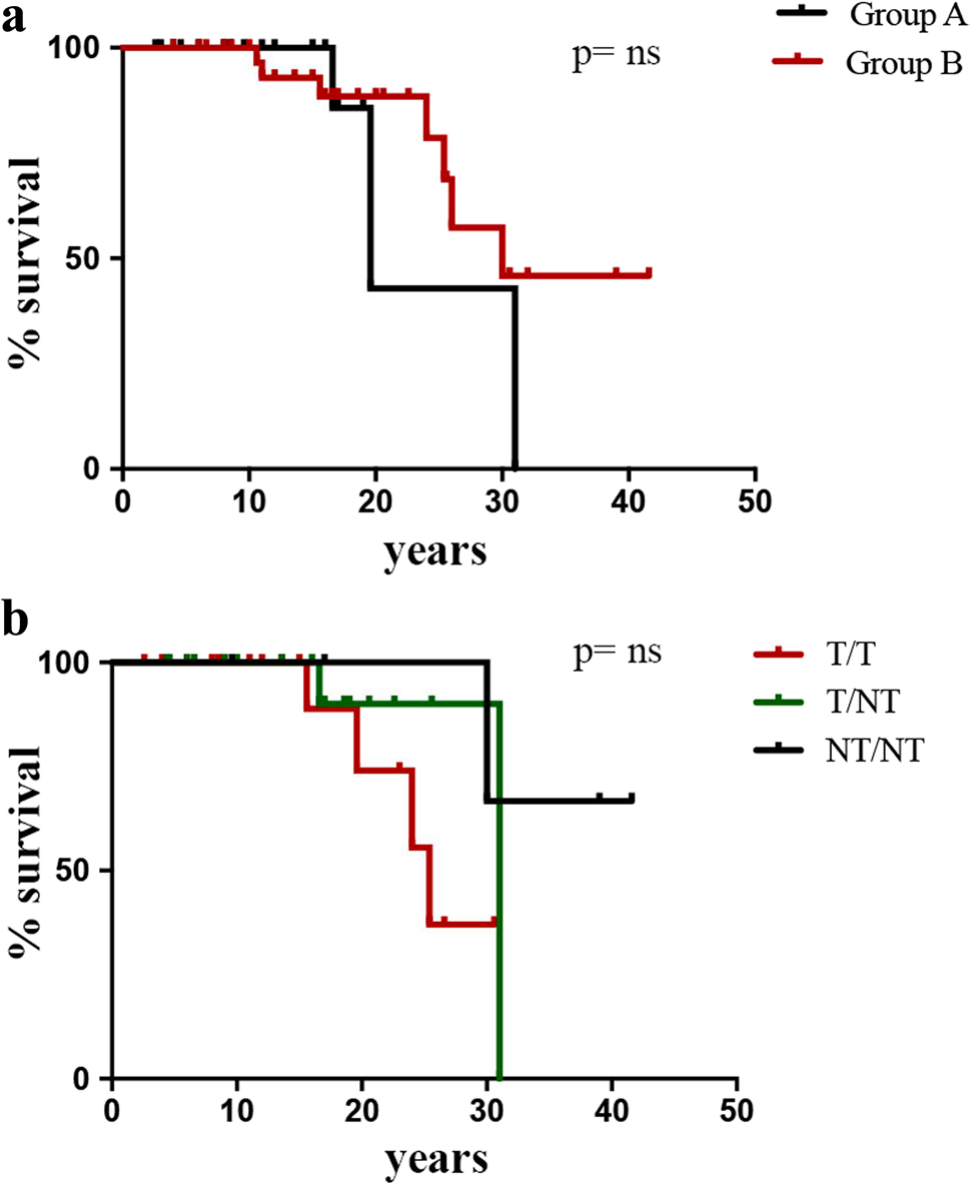
Survival rate of Italian AT cohort. **a** Comparison among lymphopenic (Group A) and non-lymphopenic patients (Group B). **b** Comparison among T/T, T/NT, and NT/NT patients. *T/T* bi-allelic truncating mutations, *T/NT* at least one non-truncating mutation, *NT/NT* bi-allelic non-truncating mutations

Survival analysis was limited by the low number of patients. As shown in Fig. 4a, patients who belonged to Group A had a higher chance to die earlier, since only 42.8% of the cohort were alive at the age of 19.6 years. Furthermore, about 63% of T/T patients died by 25 years, while, at this age, more than 80% of T/NT and NT/NT subjects were still alive. The hazard ratio between T/NT versus T/T was 2.82 (95% CI 0.47–17). Patients with both non-truncating mutations seemed to have a slightly higher, although not significant, chance of survival with a hazard ratio (HR) of 3.26 (95% CI 0.51–20.8).

## Discussion

AT is a very complex multisystem inherited disorder characterized by progressive neurological abnormalities, immunodeficiency, recurrent infections, oculocutaneous abnormalities, proneness to malignancies, and by other systemic manifestations. The prognosis is very poor since the majority of patients require wheelchair by 8–10 years of age and most patients usually die in their mid-20 s from malignancy or chronic pulmonary disease [19]. At present, no curative treatment exists and only supportive therapies or disease-modifying agents are available [11, 12, 20–23]. The AT neurological phenotype is quite variable since patients with a mild or atypical presentation have been described. Usually, these subjects have a late-onset disease, very rarely in the adulthood [24, 25]. In the present study, we performed a longitudinal analysis of immunological features of AT patients and investigated a possible genotype–phenotype correlation. Patients were categorized on the basis of their lymphocyte counts at the diagnosis and according to their genotype. We found that the disease onset was significantly earlier in lymphopenic rather than in non-lymphopenic patients. In the subgroup of lymphopenic patients Fup stops at younger ages, indirectly suggesting a lower survival associated with lymphopenia.

A wide spectrum of immunological abnormalities was observed in the whole cohort, ranging from near-normal humoral and cellular immunity to a phenotype similar to atypical severe combined immunodeficiency, according to the ESID diagnostic criteria. In keeping with our results, recently a few AT patients were identified through T (TREC) cell recombination excision circles and kappa-deleting recombination excision circle (KREC) newborn screening program [26, 27]. These patients showed profound B and T cell lymphopenia associated to decreased lymphocyte proliferation responses, similar to what observed in patients affected with severe combined immunodeficiency. Furthermore, in a cohort of Iranian patients diagnosed as CID, according to the ESID criteria, several patients were AT [28, 29].

Almost 26% of the cohort we studied had, at diagnosis, a reduction of IgG serum levels, isolated or combined with a reduction of other Ig classes, as reported in other cohorts. During the Fup 35.6% required Ig replacement therapy, with no difference according the genotype. On the contrary, only 12.3% of the subjects had high IgM levels, which is lower than what reported in the Israelian cohort [30]. Furthermore, we did not observe a progressive increase in the number of subjects with high IgM levels over time.

We did not find any statistically significant correlation between lymphopenia and genotype. However, it is noteworthy that all patients carrying both missense mutations were all non-lymphopenic at diagnosis. In addition, in 3 of them a normal proliferative response to PHA was documented, suggesting in both cases a sensitive dose effect of ATM on the immune functionality. Again, no relationship to the genotype was noted in the distribution of the T cell subsets.

T/T patients showed lower B cell counts as compared to T/NT. T/T patients also exhibited a higher number of *per-patient* infectious episodes, namely, pneumonia. This finding is consistent with the recent observation reporting abnormalities in B cell subsets, including IgM memory B cells and switched memory B cells, in AT patients with pneumonia and otitis media. Similarly, to our data, no correlation in this study was found between these complications and the other cellular subsets [31]. Previous studies on genotype–phenotype correlation in AT revealed heterogeneous results. Furthermore, phenotypic differences in siblings carrying the same *ATM* pathogenic variant have also been reported, indicating that several additional factors interfere with the disease expressivity.

We observed that the absolute number of CD3^+^ and CD8^+^ cells did not change over the time in the overall group as compared to their values at diagnosis. In contrast, a significant decrease in the absolute number of CD19^+^ cells and an increase of CD4^+^ cells was noted. In lymphopenic patients a decline of both CD4^+^ and CD19^+^ cells was noted, but classical opportunistic infections, as that from *Pneumocystis jirovecii*, did not occur in our cohort of AT patients, indicating that the quantitative deterioration of these cells does not equate a functional progressive immune deficiency. It has been reported that the loss of ATM expression in AT cells triggers a senescent-like phenotype, including upregulation of genes associated to senescence and cancer, decreased cellular replication capacity, shortening telomeres, and autophagy abnormalities [32–35]. The progressive immunological deterioration in AT patients, which parallels the neurological impairment, may account for the increased frequency or greater severity of infections with age.

When the inference of lymphocyte count on the clinical course of AT was evaluated, it was noted that cutaneous candidiasis, gastrointestinal infections, and otitis were more frequent in lymphopenic subjects, while a trend to more frequent interstitial pneumopathy and chronic bronchopneumopathy was noted in non-lymphopenic patients. In particular, consistent with other studies, we confirmed that most patients suffered from recurrent respiratory infections [36]. It should be noted that in the normal population 6–7.4% of children during the first 6–10 years of life have recurrent respiratory infections, including otitis and upper and lower airway infections [37, 38]. In our cohort of AT patients, we found a slight higher incidence of common infections of childhood, including otitis or sinusitis. However, a definitive conclusion cannot be provided since the patients enrolled in the study belonged to different age groups. Of note, over a 6-year Fup we observed a high frequency of pneumothorax (8.7% of cases) when compared to other severe chronic bronchopneumopathies, such as cystic fibrosis or patients with alpha-1 antitrypsin deficiency, where pneumothorax occurred with a frequency of 2–3% over a 10-year period [39, 40]. Although no definitive explanation may be given, pneumothorax is usually considered as the result of the recurrent pulmonary infectious/inflammatory insults and the consequence of the progressive pulmonary fibrosis. A high incidence of pleural complications, including pneumothorax, has also been reported in patients with immunodeficiency and immune dysregulation/hyperinflammation, suggesting that mechanisms related the aforementioned conditions, along with the pulmonary fibrosis, could contribute to the pathogenesis of pneumothorax in AT [41]. Little is known about the function of ATM in pulmonary epithelial cells [42]. A high bronchoalveolar sensitivity to ROS-induced DNA damage has been documented in Atm-deficient mouse model, supporting the hypothesis that ATM plays a pivotal role in the control of oxidative stress-driven lung inflammation and fibrosis [43]. In a previous study, pneumothorax has been correlated to a poor prognosis for AT patients since it does not benefit from medical or surgical therapeutic approaches, thus resulting in a high mortality rate within 6 months from its detection [44].

Previous studies reporting histologic evaluation of lung in AT patients revealed mainly infiltrates consisting of a lymphocytic or lymphohistiocytic cells, atypical epithelial and interstitial cells, and only rare neutrophils or plasma cells [44, 45]. This aspect is similar to what has been described in patients affected with common variable immunodeficiency (CVID), in whom increased chromosomal radiosensitivity, hallmark of AT, may be occasionally found [46]. Histologic studies performed in CVID patients with granulomatous-lymphocytic interstitial lung disease (GLILD) revealed infiltration of both T and B lymphocytes [47–49]. T cell dysregulation rather than lymphopenia is associated with increased levels of pro-inflammatory cytokines, which may contribute to the pathogenesis of inflammatory manifestations [50, 51].

As for the clinical manifestations of immune dysregulation, 23% of our cohort had autoimmune disorders, the more frequent organ involved being the skin with vitiligo and psoriasis. The mechanisms underlying autoimmunity in AT have not been clearly elucidated. It has been hypothesized that ineffective antibody maturation during infection, immune senescence, and inflammaging may have a role. In our patients, although there was not a statistically significant difference between the two groups, a trend to a higher incidence of autoimmune manifestations was noted among AT patients belonging to the non-lymphopenic group (Group B) suggesting a potentially expansion of senescent T cells. Cellular senescence is a process that may contribute to age-related dysfunction and chronic sterile inflammation [52]. ATM may control senescence by different pathways, including the lysosomal–mitochondrial axis, accelerated telomere shortening, and telomere fusions. Telomere length emerges as a critical regulator of T cell survival, since it may determine longevity of long-lived cells, potentially involved in the pathogenic events underlying autoimmunity. A few studies suggested that long-lived ATM-deficient phagocytes, due to persistent inflammatory stimuli, could play a role in the pathogenesis of cutaneous granulomas in AT patients [53].

Similar to what previously extensively documented in other cohorts [2], 20% of all AT patients developed cancer. The occurrence of cancer in AT patients is a well-known finding, representing the second cause of death. As shown in many studies, malignancies are strictly related to the defects of the DNA repair. In the attempt to define predictive factors, the incidence of cancer was compared between the groups. Again, a trend to a higher but not statistically significant frequency of cancer among Group B patients was noted.

A profound intrafamilial variability has been observed with respect to both immunological parameters and clinical manifestations. In our cohort, the discordance among siblings was noted either in the T cell compartment or in humoral immunity, as well as in the occurrence of cancer. These observations suggest that epigenetic factors, gene modifiers, or a redundancy of the DNA repair pathway may interfere with the phenotypic expression and variability [34, 54]. In favor of this hypothesis, it has been reported that the activation of other proteins downstream the ATM pathway, including the Mre11-Rad50-Nbs1 complex, may rescue the classical AT phenotype [55, 56].

In conclusion, the identification of patients with a higher risk to develop a malignancy or immune dysregulation and the recognition of predictive factors of worst prognosis could help manage these patients. We found that lymphopenia at diagnosis is related per se to early AO, and to a further progressive decline of either T or B cell numbers. However, it should also be mentioned that lymphopenia at the diagnosis does not necessarily imply a risk of humoral immunodeficiency. Furthermore, lymphopenic subjects have a higher chance to have bi-allelic truncating mutations. By contrast, normal lymphocyte counts at diagnosis seem to be more frequently associated to ILD, immune dysregulation, and malignancy, even though studies on larger cohorts of patients are needed. Patients carrying bi-allelic truncating mutations have a more severe B cell lymphopenia and a reduced life expectancy. Dynamic change of cellular compartment also occurs during Fup, suggesting that several mechanisms, including chronic inflammation driven by persistent genotoxic stress and accelerated telomere shortening, could be important contributor to premature immune failure in AT subjects [53, 57]. For these patients, microbiologic surveillance and respiratory monitoring is mandatory. More personalized periodic screening aimed at identifying autoimmune disorders or malignancies should be addressed in these patients.

## Supplementary Information

Below is the link to the electronic supplementary material.

Supplementary file1 (TIF 7837 KB)

Supplementary file2 (DOCX 15 KB)

## Data Availability

Data are available upon reasonable request. For further information please contact, pignata@unina.it.
