# Supplementary figures and images for: Progressive Depletion of B and T Lymphocytes in Patients with Ataxia Telangiectasia: Results of the Italian Primary Immunodeficiency Network
Source: J Clin Immunol. 2022 Mar 8;42(4):783–97. doi: 10.1007/s10875-022-01234-4 (PMC9166859; doi:10.1007/s10875-022-01234-4)

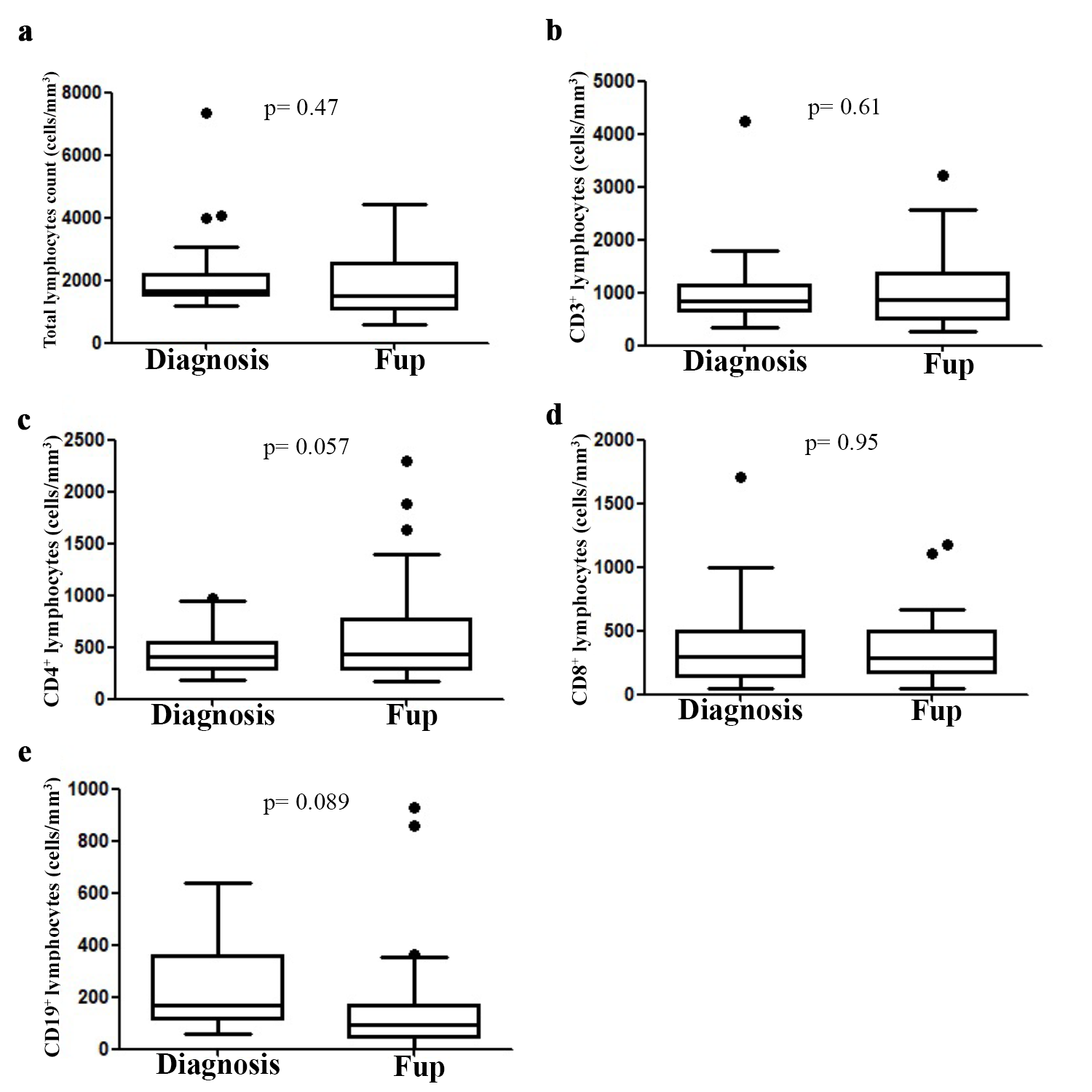

Supplement: Supplementary file 1 — Supplementary file1 (TIF 7837 KB) [file 10875_2022_1234_MOESM1_ESM.tif]
