# Supplementary material for: Progressive Depletion of B and T Lymphocytes in Patients with Ataxia Telangiectasia: Results of the Italian Primary Immunodeficiency Network
Source: J Clin Immunol. 2022 Mar 8;42(4):783–97. doi: 10.1007/s10875-022-01234-4 (PMC9166859; doi:10.1007/s10875-022-01234-4)
Supplement: Supplementary file 2 — Supplementary file2 (DOCX 15 KB) [file 10875_2022_1234_MOESM2_ESM.docx]

**Table S1** Cellular defects in the Italian AT cohort

|  | Diagnosis | T/T | T/NT-NT/NT | Unclassified | *P* value |
| --- | --- | --- | --- | --- | --- |
|  | No. (%) | No. (%) | No. (%) | No. (%) |  |
| Group A | 27 (41.5) | 13 (52) | 8 (30.8) | 6 (42.8) | 0.16 |
| Group B | 38 (58.5) | 12 (48) | 18 (69.2) | 8 (57.2) | 0.16 |
| Normal T-B- **cell counts** | 11 (17.4) | 3 (12) | 7 (26.9) | 1 (7.1) | 0.29 |
| CD3^+^ deficiency | 39 (61.9) ^ǂ^ | 17 (68) | 13 (50) | 9 (64.3) | 0.25 |
| CD4^+^ deficiency | 43 (68.2) ^ǂ^ | 18 (72) | 16 (61.5) | 9 (64.3) | 0.55 |
| CD8^+^ deficiency | 31 (49.2) ^ǂ^ | 14 (56) | 12 (46.1) | 5 (35.7) | 0.57 |
| CD19^+^ deficiency | 21 (33.3) ^ǂ^ | 10 (40) | 6 (23.1) | 5 (35.7) | 0.23 |
| **CD3^-^CD16^+^/CD56^+^** deficiency | 1 (1.5)* | 1 (4.8) | (0) | 0 (0) | / |
| **CD3^-^CD16^+^/CD56^+^** increase | 14 (31.1)* | 7 (33.3) | 6 (40) | 1 (11.1) | 0.73 |
| Proliferative response to  PHA <30% ctr | 13 (52) ^¥^ | 5 (41.6) | 6 (46.1) | 2 |  |
| PHA <10% ctr | 6 (24) ^¥^ | 3 (25) | 2 (15.4) | 1 |  |
| **PHA >30% ctr** | **12 (48) ^¥^** | **5 (41.6)** | **7 (53.8)** | **0** |  |

*T/T*, biallelic truncating mutations; *T/NT*, at least 1 non-truncating mutation; *NT/NT*, biallelic non-truncating mutations

*PHA*, Phytohemagglutin

^ǂ^data available for 63 patients (T/T: 25, T/NT: 26, of whom 5 NT/NT).

^*^data available for 45 subjects (T/T: 21, T/NT 15, of whom 4 NT/NT).

^¥^data available for 25 patients (T/T: 12, T/NT 13, of whom 3 NT/NT)

*P* value: T/T vs T/NT-NT/NT
